# Supplementary material for: Activation of transposable elements is linked to a region- and cell type–specific interferon response in Parkinson’s disease
Source: Sci Adv. 2026 Sep 2;12(36):eaed2952. doi: 10.1126/sciadv.aed2952 (PMC13537272; doi:10.1126/sciadv.aed2952)
Supplement: Supplementary file 1 [file sciadv.aed2952_sm.pdf]

Science Advances
AAAS

Supplementary Materials for

Activation of transposable elements is linked to a region- and cell type–specific interferon response in Parkinson's disease

Raquel Garza *et al.*

Corresponding author: Johan Jakobsson, johan.jakobsson@med.lu.se

Sci. Adv. **12**, eaed2952 (2026)
DOI: 10.1126/sciadv.aed2952

This PDF file includes:

Figs. S1 to S6
Tables S1 and S2

SUPPLEMENTAL MATERIAL

Supplemental Figures

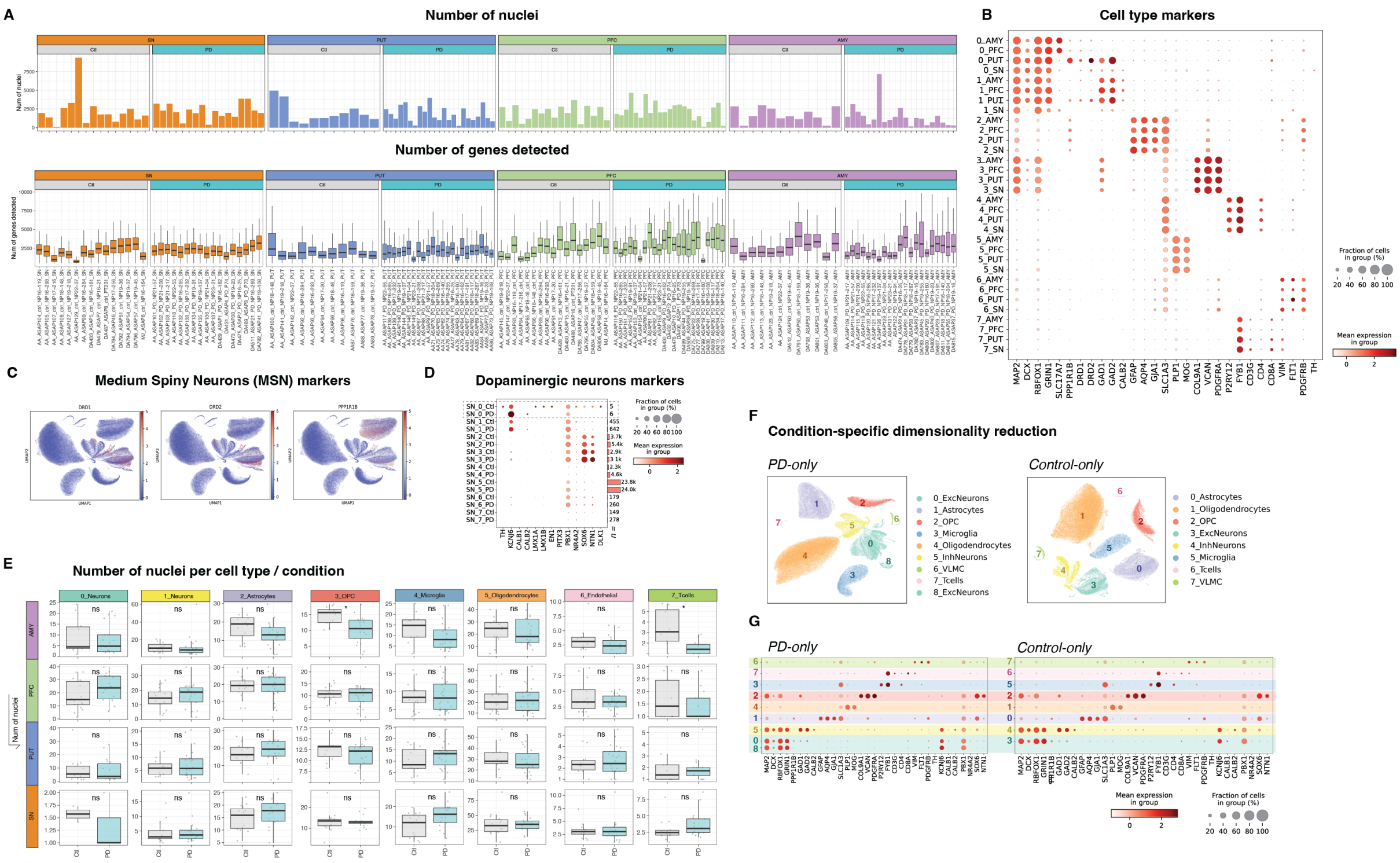

Figure S1. snRNA-seq quality control metrics, canonical markers gene expression, comparison of cell type composition between PD and control, and condition-specific UMAP visualizations. A) *Number of nuclei (top) and genes detected (bottom) in each sample.* ***B)*** *Dotplot showing canonical gene markers in each cluster and region. Dot size corresponding to the fraction of nuclei in cluster expressing the marker. Color representing average gene expression in cluster.* ***C)*** *UMAPs coloured by normalized expression of MSN gene markers.* ***D)*** *Dotplot showing canonical dopaminergic neuron markers in each cluster in the SN. Dot size corresponding to the fraction of nuclei in cluster expressing the marker. Color representing average gene expression in cluster.* ***E)*** *Boxplot showing the number of nuclei per individual in each cluster (y-axis: squared root of the number of nuclei per sample) grouped by condition. T-tests showing a statistically significant difference in the number of nuclei in each cluster.* ***F)*** *UMAP showing unbiased clustering of all nuclei from PD (left; 9 clusters) and Control (right; 8 clusters) individuals. Clusters are colored by cell type.* ***G)*** *Dotplot showing canonical gene markers expression from PD (left) and Control (right) clusters. Dot size corresponding to the fraction of nuclei in cluster expressing the marker. Color representing average gene expression in cluster.*

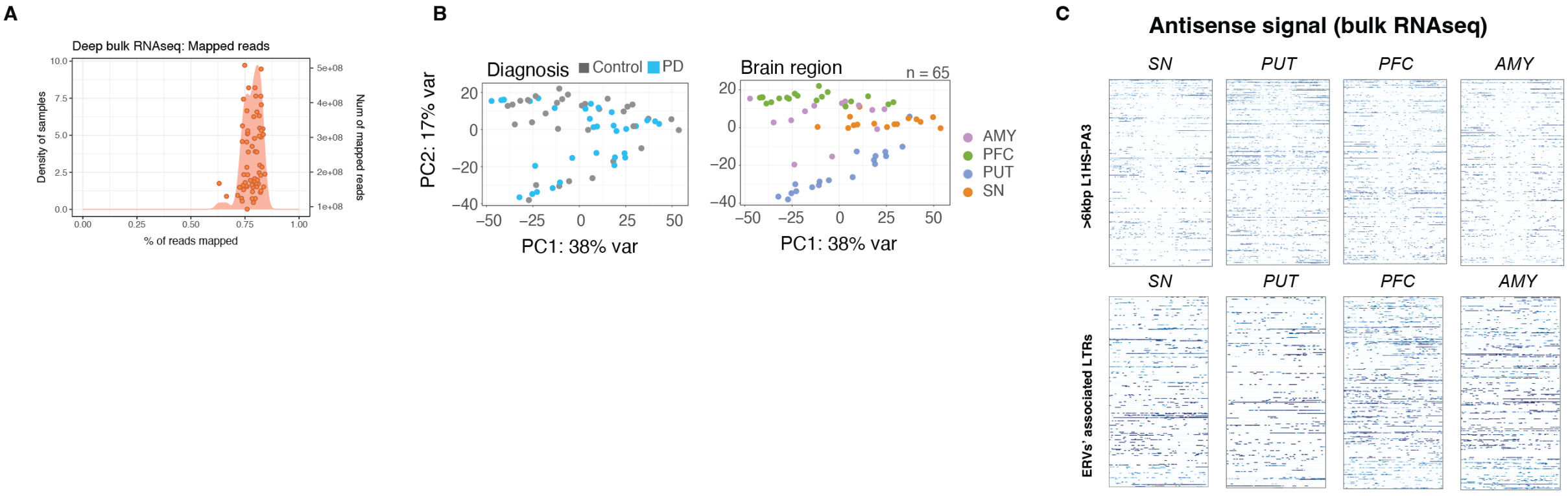

Figure S2. Deep bulk RNA-seq quality control graphs. A) *Distribution of number of reads and percentage of reads mapped to the genome among the deep bulk RNA-seq samples.* ***B)*** *Principal component analysis of the samples sequenced with deep bulk RNA-seq plotted by diagnosis (top: Diagnosis) or brain region (bottom: Brain region). AMY= amygdala; PFC= prefrontal cortex; PUT= putamen; SN= substantia nigra* ***C)*** *Top: Antisense expression heatmap showing binned signal (10bp) over expressed >6kbp L1HS-L1PA3 elements (from Figure 2b, n = 520) with 6kbp windows up and downstream. Elements are scaled to 6kbp. Color represents log2 RPKM values. Bottom: Antisense expression heatmap showing binned signal (10bp) over the associated LTRs to the expressed ERV elements (from Figure 2b, n = 739) with 5kbp windows up and downstream. Elements are scaled to 1kbp. Color represents log2 RPKM values.*

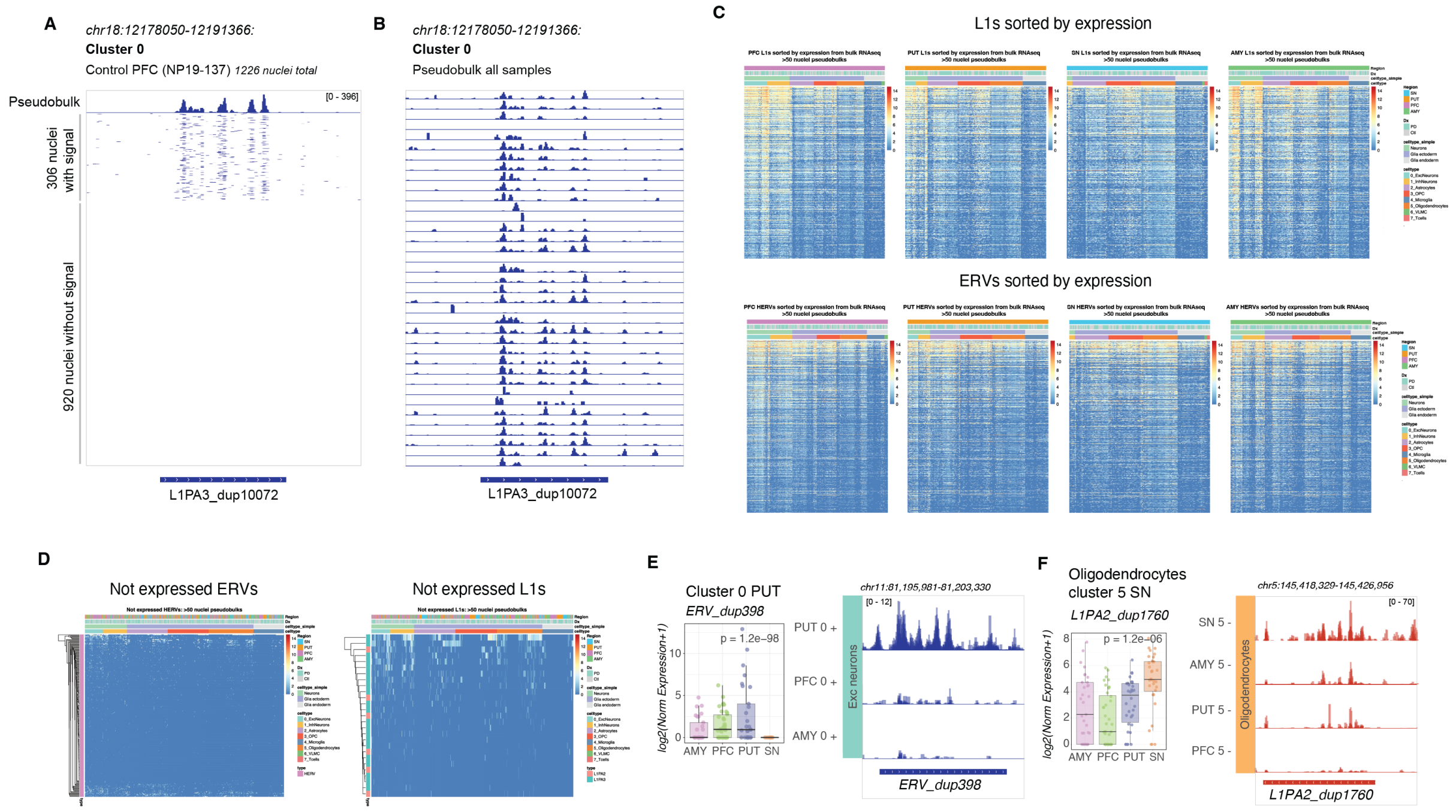

Figure S3. Validation analyses of cell-type specific TE expression from snRNA-seq. A) *Genome browser tracks showing, at the top, the pseudobulk of a single sample (cluster 0 of NP19-137 PFC, normalized by gene size factors), followed by 1226 individual nucleus tracks contributing to the pseudobulk (normalized by RPKM). Positive-strand transcription is shown.* ***B)*** *Genome browser tracks showing all cluster 0 pseudobulks across samples. Tracks were normalized by gene size factors, positive-strand transcription is shown.* ***C)*** *Heatmaps showing cell-type (columns) TE (rows) expression in the different brain regions. ERVs (left) and >6kbp L1HS-L1PA3 (right) shown were found not to be expressed in the deep-bulk RNAseq. Column annotation showing cell-types, condition (Dx, PD or Ctl), and brain region.* ***D)*** *Heatmaps showing cell-type (columns) TE (rows) expression in the different brain regions. ERVs (left) and >6kbp L1HS-L1PA3 (right) ordered by expression in deep-bulk RNAseq from the respective brain region. Column annotation showing cell-types, condition (Dx, PD or Ctl), and brain region.* ***E)*** *Example of an excitatory neuron-, putamen-specific ERV. To the left, boxplot showing samples' cell-type expression (DESeq2, Wald test, normalized by gene size factors). To the right, genome browser tracks showing excitatory neurons in PUT, AMY and PUT (normalized by gene size factors).* ***F)*** *Example of an oligodendrocyte-, SN-specific L1PA2. To the left, boxplot showing cell-type expression (DESeq2, Wald test, normalized by gene size factors). To the right, genome browser tracks showing excitatory neurons in PUT, AMY and PUT (normalized by gene size factors). Genome tracks in blue showing transcription from the positive strand, red showing transcription from the negative strand.*

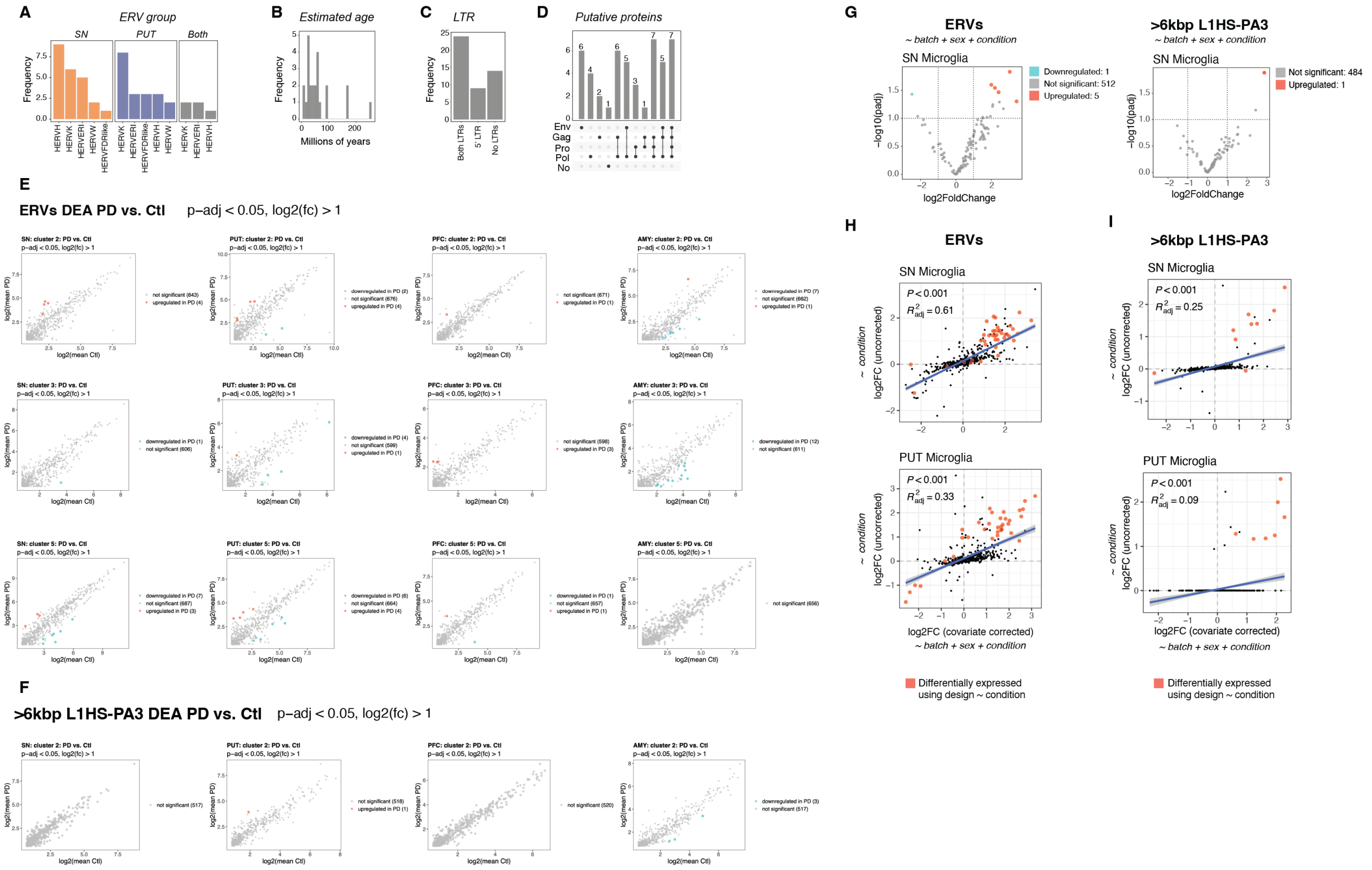

Figure S4. Classification of ERVs upregulated in PD microglia (SN and PUT), differential expression analysis (DEA) of ERVs and L1s in the remaining cell types, and validation of TE DEA results with covariate correction. A) *Subfamilies of upregulated ERVs in SN and PUT microglia.* ***B)*** *Estimated evolutionary age of upregulated ERVs in PD SN and PUT microglia.* ***C)*** *Presence of LTRs in upregulated ERVs in PD SN and PUT microglia.* ***D)*** *Putative proteins as reported by retrotector in upregulated ERVs in PD SN and PUT microglia.* ***E)*** *Mean plots showing ERV differential expression analysis results in the cell types not shown in main figure for each brain region (red indicating TEs of padj < 0.05 & log2FC > 1. DESeq2 Wald-test).* ***F)*** *Mean plots showing >6kbp L1HS-PA3 differential expression analysis results in the cell types not shown in main figure for each brain region (red indicating TEs of padj < 0.05 & log2FC > 1. DESeq2 Wald-test).* ***G)*** *Volcano plot showing ERV (left) and >6kbp L1HS-PA3 (right) DEA in SN microglia using design ~ batch + sex + condition (red indicating TEs of padj < 0.05 & log2FC > 1. DESeq2 Wald-test).* ***H-I)*** *Scatter plots showing correlation between log2FC calculated with (x-axis) and without (y-axis) covariate correction. Red indicates TEs of padj < 0.05 & |log2FC| > 1 using uncorrected test (TE log2FC and padj calculated by DESeq2 Wald-test. Fitted linear models using method = lm, ggpmisc::stat_poly_line).*

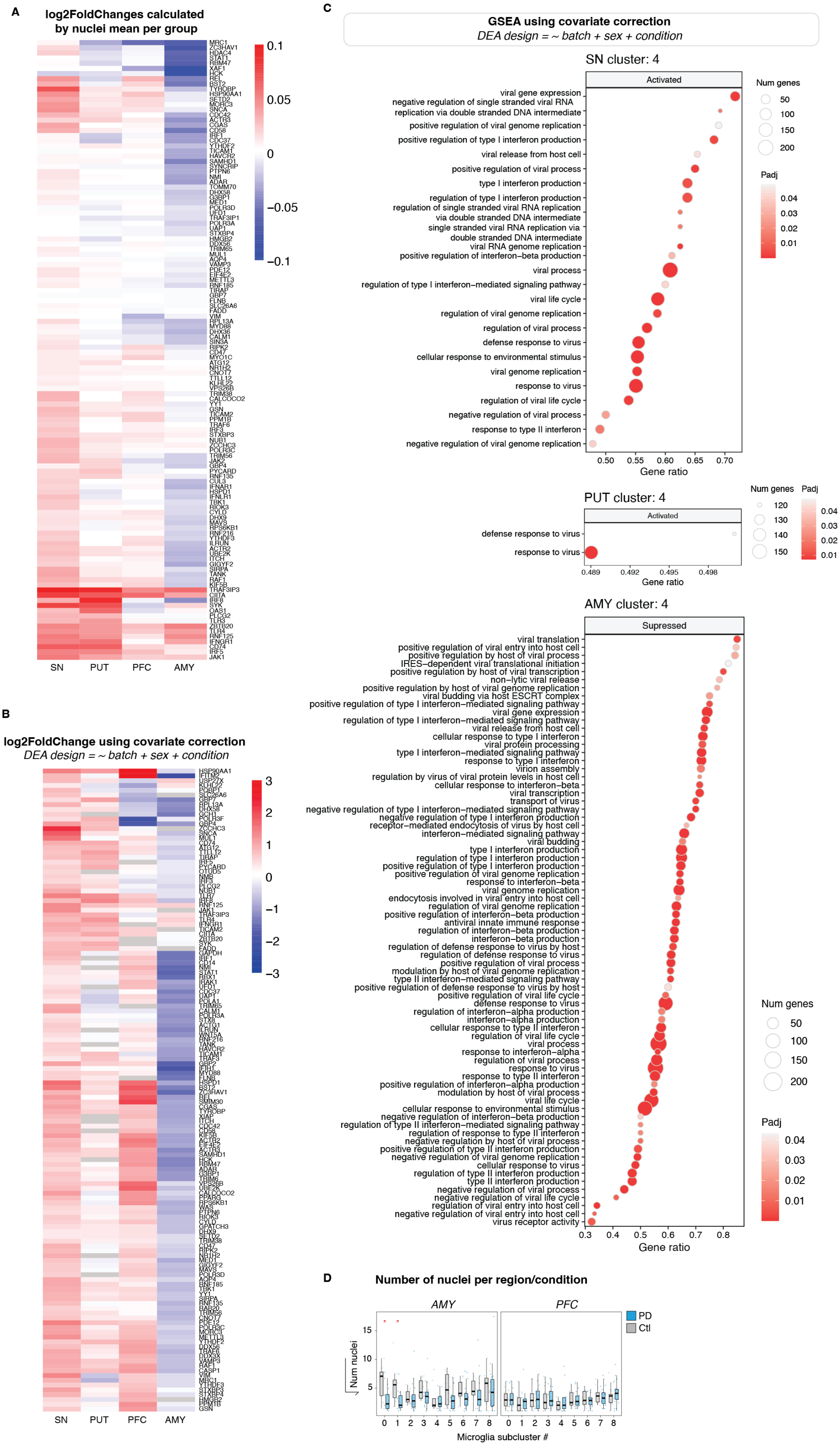
A
log2FoldChanges calculated
by nuclei mean per group
0.1
0.05
0
−0.05
−0.1
SN
PUT
PFC
AMY
B
log2FoldChange using covariate correction
DEA design = ~ batch + sex + condition
3
2
1
0
−1
−2
−3
SN
PUT
PFC
AMY
C
GSEA using covariate correction
DEA design = ~ batch + sex + condition
SN cluster: 4
Activated
viral gene expression
negative regulation of single stranded viral RNA replication via double stranded DNA intermediate
positive regulation of viral genome replication
positive regulation of type I interferon production
viral release from host cell
positive regulation of viral process
type I interferon production
regulation of type I interferon production
regulation of single stranded viral RNA replication via double stranded DNA intermediate
single stranded viral RNA replication via double stranded DNA intermediate
viral RNA genome replication
positive regulation of interferon−beta production
viral process
regulation of type I interferon−mediated signaling pathway
viral life cycle
regulation of viral genome replication
regulation of viral process
defense response to virus
cellular response to environmental stimulus
viral genome replication
response to virus
regulation of viral life cycle
negative regulation of viral process
response to type II interferon
negative regulation of viral genome replication
0.50
0.55
0.60
0.65
0.70
Gene ratio
Num genes
50
100
150
200
Padj
0.04
0.03
0.02
0.01
PUT cluster: 4
Activated
defense response to virus
response to virus
0.489
0.492
0.495
0.498
Gene ratio
Num genes
120
130
140
150
Padj
0.04
0.03
0.02
0.01
AMY cluster: 4
Supressed
viral translation
positive regulation of viral entry into host cell
positive regulation by host of viral process
IRES−dependent viral translational initiation
positive regulation by host of viral transcription
non−lytic viral release
positive regulation by host of viral genome replication
viral budding via host ESCRT complex
positive regulation of type I interferon−mediated signaling pathway
viral gene expression
regulation of type I interferon−mediated signaling pathway
viral release from host cell
cellular response to type I interferon
viral protein processing
type I interferon−mediated signaling pathway
response to type I interferon
virion assembly
regulation by virus of viral protein levels in host cell
cellular response to interferon−beta
viral transcription
transport of virus
negative regulation of type I interferon−mediated signaling pathway
negative regulation of type I interferon production
receptor−mediated endocytosis of virus by host cell
interferon−mediated signaling pathway
viral budding
type I interferon production
regulation of type I interferon production
positive regulation of type I interferon production
positive regulation of viral genome replication
response to interferon−beta
viral genome replication
endocytosis involved in viral entry into host cell
regulation of viral genome replication
positive regulation of interferon−beta production
antiviral innate immune response
regulation of interferon−beta production
interferon−beta production
regulation of defense response to virus by host
regulation of defense response to virus
positive regulation of viral process
modulation by host of viral genome replication
type II interferon−mediated signaling pathway
positive regulation of defense response to virus by host
positive regulation of viral life cycle
defense response to virus
regulation of interferon−alpha production
interferon−alpha production
cellular response to type II interferon
regulation of viral life cycle
viral process
response to interferon−alpha
regulation of viral process
response to virus
response to type II interferon
positive regulation of interferon−alpha production
modulation by host of viral process
viral life cycle
cellular response to environmental stimulus
negative regulation of interferon−beta production
regulation of type II interferon−mediated signaling pathway
regulation of response to type II interferon
negative regulation by host of viral process
positive regulation of type II interferon production
negative regulation of viral genome replication
cellular response to virus
regulation of type II interferon production
type II interferon production
negative regulation of viral process
negative regulation of viral life cycle
regulation of viral entry into host cell
negative regulation of viral entry into host cell
virus receptor activity
0.3
0.4
0.5
0.6
0.7
0.8
Gene ratio
Num genes
50
100
150
200
Padj
0.04
0.03
0.02
0.01
D
Number of nuclei per region/condition
AMY
PFC
PD
Ctl
15
10
5
√ Num nuclei
0 1 2 3 4 5 6 7 8
0 1 2 3 4 5 6 7 8
Microglia subcluster #

Figure S5. Validation of gene DEA results using group means, and covariate correction. A) *Heatmap coloured by stable log2FC (scaled mean in PD vs scaled mean in Control; see methods "Validation of differential expression analyses") of the genes identified as the core enrichment of the GO terms shown in Figure 6.* ***B)*** *Heatmap of genes identified as the core enrichment of the GO terms shown in Figure 6 coloured by log2FC in microglia using covariate corrected data (DESeq2, design = ~ batch + sex + condition).* ***C)*** *Interferon and viral related GO terms selected from the results of a gene set enrichment analysis (GSEA) of SN (left), PUT (middle), and AMY (right) in PD Microglia (clusterProfiler::gseGO) using covariate correction (DESeq2, design = ~ batch + sex + condition).* ***D)*** *Boxplot showing the number of nuclei per individual in each microglia subcluster (y-axis: squared root of the number of nuclei). T-tests showing a statistically significant difference in the number of microglia in AMY (clusters 0 and 1).*

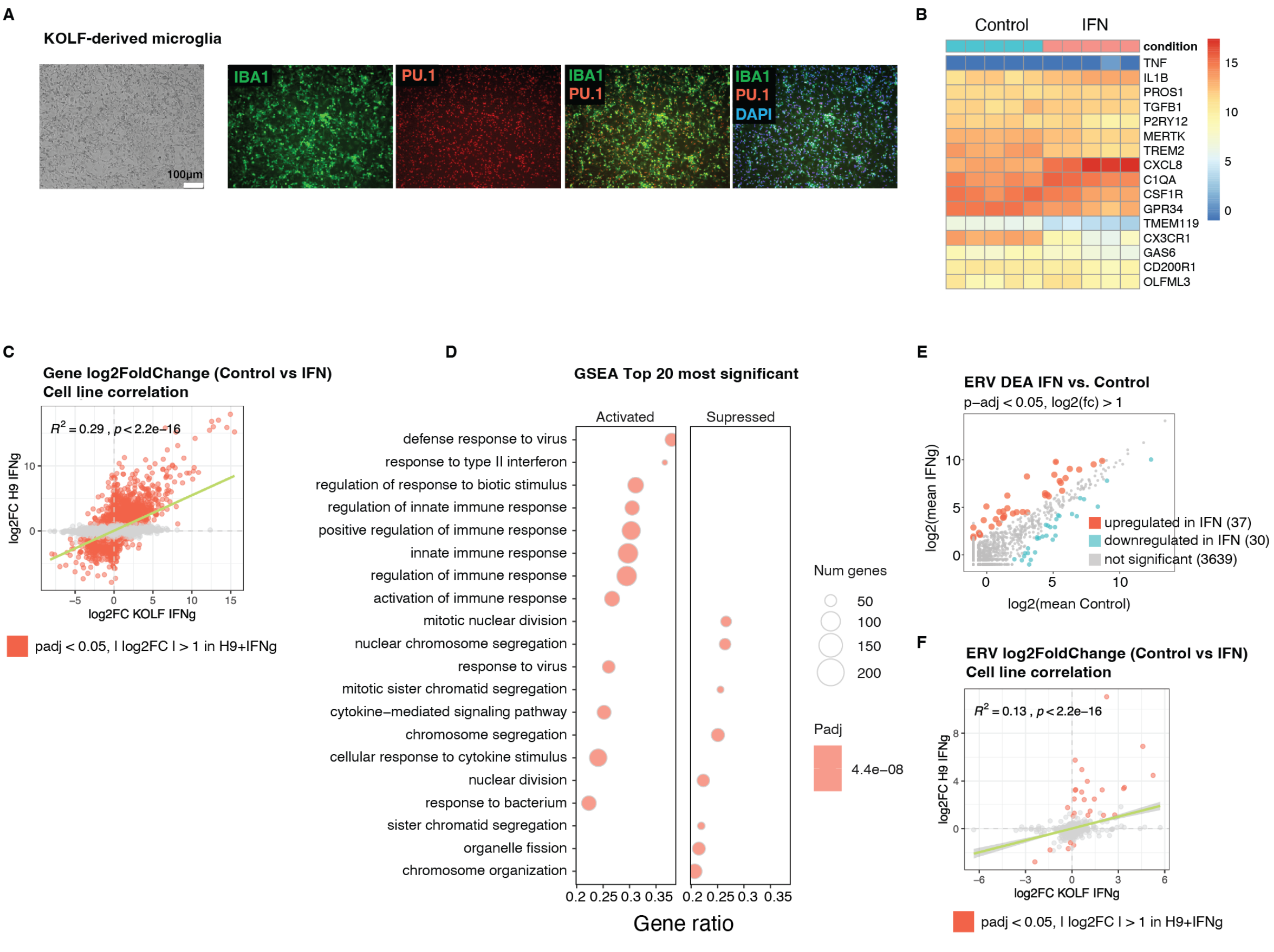

Figure S6. Validation of in vitro findings using hiPSC-derived microglia from an independent cell line (KOLF). A) *Left: Brightfield image showing KOLF hiPSC-derived microglia at 10 days of in vitro differentiation. Scale bar = 100µm. Right: Immunostaining of microglial markers (PU.1, and IBA-1). Scale bar = 100µm.* ***B)*** *Heatmap showing microglial marker expression from bulk RNA sequencing.* ***C)*** *Scatter plots showing correlation between genes' log2FC (Untreated vs IFNg treated microglia) in H9-derived microglia (y-axis) and KOLF-derived microglia (x-axis). Red indicates differentially expressed genes in H9-derived microglia (padj < 0.05 & | log2FC | > 1; DESeq2 Wald-test. Fitted linear model using method = lm, ggpmisc::stat_poly_line).* ***D)*** *Gene set enrichment analysis (GSEA) results of Untreated vs IFN treated KOLF-derived microglia.* ***E)*** *Mean plot showing ERV differential expression analysis results in KOLF-derived microglia (red indicating ERVs of pdj < 0.05 & log2FC >*

1. DESeq2 Wald-test). ***F)*** *Scatter plots showing correlation between ERVs log2FC (Untreated vs IFNg treated microglia) in H9-derived microglia (y-axis) and KOLF-derived microglia (x-axis). Red indicates differentially expressed ERVs in H9-derived microglia (padj < 0.05 & | log2FC | > 1; DESeq2 Wald-test. Fitted linear model using method = lm, ggpmisc::stat_poly_line).*

Supplemental Tables

Table S1. Datasets and donors' information for the post-mortem cohort. *Datasets and donors' information from the 46 individuals within the post-mortem cohort. Information includes the condition (control or Parkinson's disease), brain region (PFC= prefrontal cortex; PUT= putamen; SN= substantia nigra; AMY= amygdala), age, sex (M= male; F= female), post-mortem interval (PMI) and Braak stage.*

Sample ID	Diagnosis	Region	Age	Sex	PMI	Braak	10x snRNAseq	Deep Bulk RNAseq
NP16-00119	Control	PFC	74	M	74	0	Yes	Yes
NP16-00119	Control	PUT	74	M	74	0	Yes	Yes
NP16-00119	Control	SN	74	M	74	0	Yes	Yes
NP16-00119	Control	AMY	74	M	32	0	Yes	Yes
NP16-00284	Control	PFC	70	F	32	0	Yes	Yes
NP16-00284	Control	PUT	70	F	32	0	Yes	Yes
NP16-00284	Control	SN	70	F	32	0	Yes	Yes
NP16-00284	Control	AMY	70	F	32	0	Yes	Yes
NP16-00285	PD	AMY	87	M	68	5	Yes	Yes
NP16-00285	PD	SN	87	M	68	5	Yes	Yes
NP16-00285	PD	PUT	87	M	68	5	Yes	Yes
NP16-00285	PD	PFC	87	M	68	5	Yes	Yes
NP16-00293	Control	PFC	75	F	92	0	Yes	Yes
NP16-00293	Control	PUT	75	F	92	0	Yes	Yes
NP16-00293	Control	SN	75	F	92	0	Yes	Yes
NP16-00293	Control	AMY	75	F	32	0	Yes	Yes
NP16-140	PD	AMY	71	M	32	6	Yes	
NP16-140	PD	PFC	71	M	32	6	Yes	
NP16-140	PD	PUT	71	M	32	6	Yes	
NP16-160	PD	PFC	88	M	32	6	Yes	
NP16-160	PD	AMY	88	M	32	6	Yes	
NP16-160	PD	PUT	88	M	32	6	Yes	
NP16-161	Control	PFC	72	M	32	0	Yes	
NP16-161	Control	SN	72	M	32	0	Yes	
NP16-162	PD	PUT	74	M	32	6	Yes	
NP16-162	PD	SN	74	M	32	6	Yes	
NP16-162	PD	PFC	74	M	32	6	Yes	
NP16-164	Control	PFC	75	M	32	0	Yes	
NP16-164	Control	SN	75	M	32	0	Yes	
NP16-21	Control	SN	83	M	32	0	Yes	

NP16-21	Control	PFC	83	M	32	0	Yes	
NP16-25	PD	PUT	85	M	32	4	Yes	
NP16-25	PD	AMY	85	M	32	4	Yes	
NP16-25	PD	SN	85	M	32	4	Yes	
NP16-25	PD	PFC	85	M	32	4	Yes	
NP16-269	PD	PFC	73	M	32	5	Yes	
NP16-269	PD	PUT	73	M	32	5	Yes	
NP16-269	PD	SN	73	M	32	5	Yes	
NP17-00020	Control	PFC	87	M	49	0	Yes	Yes
NP17-00020	Control	PUT	87	M	49	0	Yes	Yes
NP17-00020	Control	AMY	87	M	32	0		Yes
NP17-00216	Control	PFC	77	F	59	0	Yes	Yes
NP17-00216	Control	PUT	77	F	59	0		Yes
NP17-00216	Control	SN	77	F	32	0	Yes	Yes
NP17-00232	PD	AMY	71	F	54	4	Yes	Yes
NP17-00232	PD	SN	71	F	54	4	Yes	Yes
NP17-00232	PD	PUT	71	F	54	4	Yes	Yes
NP17-00232	PD	PFC	71	F	54	4	Yes	Yes
NP17-191	PD	PFC	73	M	32	6	Yes	
NP17-191	PD	AMY	73	M	32	6	Yes	
NP17-191	PD	PUT	73	M	32	6	Yes	
NP17-256	Control	SN	72	F	32	0	Yes	
NP17-256	Control	PFC	72	F	32	0	Yes	
NP17-256	Control	AMY	72	F	32	0	Yes	
NP18-00046	Control	PFC	86	F	67	0	Yes	Yes
NP18-00046	Control	PUT	86	F	32	0	Yes	Yes
NP18-00148	Control	PFC	88	F	28	0	Yes	Yes
NP18-00148	Control	PUT	88	F	28	0	Yes	Yes
NP18-00148	Control	SN	88	F	28	0	Yes	Yes
NP18-00148	Control	AMY	88	F	28	0	Yes	Yes
NP18-117	PD	AMY	90	M	32	5	Yes	
NP18-117	PD	PFC	90	M	32	5	Yes	
NP18-117	PD	PUT	90	M	32	5	Yes	
NP18-159	Control	AMY	65	F	32	0	Yes	
NP18-159	Control	PFC	65	F	32	0	Yes	
NP18-159	Control	PUT	65	F	32	0	Yes	
NP18-287	PD	AMY	77	M	32	5	Yes	
NP18-287	PD	PUT	77	M	32	5	Yes	
NP18-304	PD	PFC	89	M	32	4	Yes	
NP18-304	PD	AMY	89	M	32	4	Yes	
NP18-304	PD	PUT	89	M	32	4	Yes	
NP19-00091	PD	AMY	82	F	67	5	Yes	Yes
NP19-00091	PD	SN	82	F	67	5	Yes	Yes

NP19-00091	PD	PUT	82	F	67	5	Yes	Yes
NP19-00091	PD	PFC	82	F	32	5	Yes	Yes
NP19-00137	PD	AMY	77	M	59	5	Yes	Yes
NP19-00137	PD	SN	77	M	59	5	Yes	Yes
NP19-00137	PD	PUT	77	M	59	5	Yes	Yes
NP19-00137	PD	PFC	77	M	59	5	Yes	Yes
NP19-00218	Control	AMY	88	M	18	0	Yes	Yes
NP19-00218	Control	SN	88	M	18	0	Yes	Yes
NP19-00218	Control	PUT	88	M	18	0	Yes	Yes
NP19-00218	Control	PFC	88	M	18	0	Yes	Yes
NP19-108	PD	SN	86	F	32	6	Yes	
NP19-108	PD	PFC	86	F	32	6	Yes	
NP19-108	PD	PUT	86	F	32	6	Yes	
NP19-16	PD	AMY	84	M	32	4	Yes	
NP19-16	PD	PUT	84	M	32	4	Yes	
NP19-16	PD	PFC	84	M	32	4	Yes	
NP19-23	PD	AMY	78	F	32	5	Yes	
NP19-23	PD	PUT	78	F	32	5	Yes	
NP19-23	PD	SN	78	F	32	5	Yes	
NP19-255	PD	AMY	76	M	32	5	Yes	
NP19-255	PD	PUT	76	M	32	5	Yes	
NP19-255	PD	PFC	76	M	32	5	Yes	
NP19-36	Control	SN	39	M	32	0	Yes	
NP19-36	Control	AMY	39	M	32	0	Yes	
NP19-36	Control	PUT	39	M	32	0	Yes	
NP19-37	Control	SN	73	M	32	0	Yes	
NP19-37	Control	PFC	73	M	32	0	Yes	
NP19-37	Control	AMY	73	M	32	0	Yes	
NP19-37	Control	PUT	73	M	32	0	Yes	
NP19-45	Control	SN	62	M	32	0	Yes	
NP19-45	Control	PFC	62	M	32	0	Yes	
NP19-45	Control	AMY	62	M	32	0	Yes	
NP21-00004	PD	AMY	75	M	48	6	Yes	Yes
NP21-00004	PD	SN	75	M	48	6	Yes	Yes
NP21-00004	PD	PUT	75	M	48	6	Yes	Yes
NP21-00004	PD	PFC	75	M	48	6	Yes	Yes
NP21-00057	PD	PFC	83	F	28	4	Yes	Yes
NP21-00057	PD	PUT	83	F	28	4	Yes	Yes
NP21-00057	PD	SN	83	F	28	4	Yes	Yes
NP21-00057	PD	AMY	83	F	32	4	Yes	Yes
NP21-00208	PD	PFC	81	M	87	6	Yes	Yes
NP21-00208	PD	PUT	81	M	87	6	Yes	Yes
NP21-00208	PD	SN	81	M	87	6	Yes	Yes

NP21-00208	PD	AMY	81	M	87	6	Yes	Yes
NP21-00217	PD	PFC	84	F	43	6	Yes	Yes
NP21-00217	PD	PUT	84	F	43	6	Yes	Yes
NP21-00217	PD	SN	84	F	43	6	Yes	Yes
NP21-00217	PD	AMY	84	F	32	6	Yes	Yes
NP22-00037	Control	AMY	90	M	56	0	Yes	Yes
NP22-00037	Control	SN	90	M	56	0	Yes	Yes
NP22-00037	Control	PUT	90	M	56	0	Yes	Yes
NP22-00037	Control	PFC	90	M	56	0	Yes	Yes
NP22-00055	PD	PFC	76	F	59	6	Yes	Yes
NP22-00055	PD	PUT	76	F	59	6	Yes	Yes
NP22-00055	PD	SN	76	F	59	6	Yes	Yes
NP22-00055	PD	AMY	76	F	59	6	Yes	Yes
NP22-00075	Control	AMY	74	M	115	0	No - hypoxic	Yes
NP22-00075	Control	SN	74	M	115	0	No - hypoxic	Yes
NP22-00075	Control	PUT	74	M	115	0	No - hypoxic	Yes
NP22-00075	Control	PFC	74	M	115	0	No - hypoxic	Yes
NP23-00021	PD	AMY	71	M	114	5	Yes	Yes
NP23-00021	PD	SN	71	M	114	5	Yes	Yes
NP23-00021	PD	PUT	71	M	114	5	Yes	Yes
NP23-00021	PD	PFC	71	M	32	5	Yes	Yes
P73	PD	SN	92	M	32	5	Yes	
P73	PD	PFC	92	M	32	5	Yes	
P74	PD	SN	63	M	32	4	Yes	
P74	PD	PFC	63	M	32	4	Yes	
PT231	Control	SN	84	M	32	0	Yes	
PT231	Control	PFC	84	M	32	0	Yes	

Table S2. Mean variance explained by individual covariates. *Mean variance explained by individual covariates (design = ~ batch + sex + condition) using all features (HERVs, L1s, and genes) as calculated by fitExtractVarPartModel (variancePartition).*

Pseudobulk (region_cluster)	Mean variance from sequencing batch	Mean variance from sex
AMY_0	0,39231496	0,1076047
AMY_1	0,41895756	0,06247276
AMY_2	0,21175977	0,04571372
AMY_3	0,28960846	0,04988992
AMY_4	0,32576012	0,09084974
AMY_5	0,13742857	0,06148692
PFC_0	0,31311396	0,05723861
PFC_1	0,31918144	0,03531423
PFC_2	0,23974501	0,02845113
PFC_3	0,42109326	0,06623028
PFC_4	0,53282174	0,15830616
PFC_5	0,22311288	0,05055917
PUT_2	0,1813245	0,0343387
PUT_3	0,17805116	0,03907945
PUT_4	0,25637325	0,05367004
PUT_5	0,13092601	0,02670692
SN_2	0,18091526	0,04250906
SN_3	0,2640864	0,05187649
SN_4	0,20861184	0,0481163
SN_5	0,17807142	0,03279578
